# Authorship, plagiarism, and copyright transfer in the scientific universe

**DOI:** 10.6061/clinics/2019/e1312

**Published:** 2019-07-22

**Authors:** Sigmar de Mello Rode, Pedro Rogério Camargos Pennisi, Thiago Leite Beaini, Janaina Paiva Curi, Sérgio Vitorino Cardoso, Luiz Renato Paranhos

**Affiliations:** I Departamento de Materiais Odontologicos e Protese, Instituto de Ciencia e Tecnologia, Universidade Estadual Paulista Julio de Mesquita Filho, Campus Sao Jose dos Campos, Sao Jose dos Campos, SP, BR; II Faculdade de Odontologia, Universidade Federal de Uberlandia, Uberlandia, MG, BR; III Faculdade de Odontologia, Universidade de Sao Paulo, Sao Paulo, SP, BR

The academic practices that result in the definition of the authorship of a scientific paper are perpetuated according to the customs or historical vision of those involved. Given the potential and substantial benefits of authorship, the improper and “unethical” inclusion of authors may seem beneficial when compared to the risk of retaliation. In fact, the hierarchy and the desire of those involved in having a work published with advisors and references in the field make such behavior common in the scientific environment. Another problem lies in the definition of the order of authors, as it does not reflect the acknowledgment each one should receive during the publication of results and may simply result from the fact that there are no widely accepted criteria for this definition.

According to the International Committee of Medical Journal Editors (ICMJE) 1, the authorship of scientific papers should meet the following conditions: 1) substantial contribution to design and planning, data acquisition, or data analysis and interpretation; 2) writing and drafting of the article or the major critical revision of its intellectual content; and 3) approving the final version for publication.

This definition implies that each author should have full knowledge of the part produced and responsibility for the information he or she provided, with the additional ability to define the responsibility of each coauthor of the publication 2. Therefore, individuals who meet these four requirements should be defined as authors. Furthermore, the corresponding author is the one who assumes most of the responsibility for contacting the journal in the process of submission and response to questions 1.

The Consortia Advancing Standards in Research Administration Information (CASRAI), through the initiative entitled Contributor Roles Taxonomy (CRediT), presents 14 attributions that may grant researchers the right of authorship of a scientific paper, namely, research design, collection and formal analysis of data, acquisition of resources, research performance, methodology development, project management, resources, software production, supervision, validation, preparation of visual resources, drafting and review, and conclusion of the written work. It is recommended that all collaborators are listed and that their actions are evaluated according to such roles 2.

Presenting the same objective, the initiative of the Committee on Publication Ethics (COPE) is intended to create a conducive environment for ethics in scientific publications. Hence, the committee created the PEST analysis that considers activities in the political, economic, social, and technological fields. The aim is to stimulate the creation of common legislation among countries, implement fundraising that will reduce competition among researchers, and promote debates in the social field through the efficient communication provided by technology 3.

Even with these parameters, there are very common situations in the academic environment, such as guest authorship (coauthorship), which occurs when the author is included in the publication without having participated in the execution of the project. Another condition is pressured authorship, or coauthorship, which occurs when the names of individuals are included in every work by members of their research team. Gifted authorship, in turn, is when the individual has a high rank 1 but has not contributed significantly to the study.

There are also situations in which advisors include their students in their work so that the students are more likely to excel in selection processes, scholarship exams, the *Sucupira* platform from the Brazilian Coordination for the Improvement of Higher Education Personnel (CAPES), and other processes. Thus, the “irregular” inclusion of authors may also harm the other authors who actually participated in the work, where they have an objective that requires the authorship order.

Brazilian Law 9610/98 4 establishes:

“The co-authorship of the work is attributed to those in whose name, pseudonym, or conventional sign it is used. Paragraph 1. It is not considered a co-author the individual who simply assisted the author in producing the literary, artistic, or scientific work by revising it, updating it, and supervising or directing its editing or presentation by any means.”

The legislation pertains to the selection of authors and coauthors of a work, which should be respected at the time of the submission of a particular publication. Thus, the ICMJE criteria 1 are reinforced; these affirm that those who participated only through funding, administrative assistance, statistical analysis, training, linguistic revision, and translations should not be considered eligible for authorship. The acknowledgment section is reserved for such participants, which is very common and should be more valued in scientific articles.

Thus, the practice of improper authorship, which is apparently irrelevant, generates injustices 5, especially in processes for research funding or academic promotions, and it contradicts one of the principles of scientific integrity: transparency. In view of the information above, it is evident that the inclusion of authors for other reasons, that do not include real participation in the writing and development of the publication, is not approved by the scientific community and may harm the professional trajectory of those who engage in such an act. Finally, as other authors have stated 6, we support the idea that guest, pressured, or ghost authorships should be opposed.

The growing need for science generates a greater number of quality articles, and coauthorship is a way to legitimize science and ensure the continuity of the scientist. Scientific partnerships reduce costs and optimize the time and use of human resources while favoring a multicentric and multidisciplinary vision by exchanging experiences and new solutions, which has encouraged a substantial increase in the number of authors per work over the last decade 6.

It should be noted that all authors have a legal responsibility regarding the information added to the manuscript 7, and they should be able to describe their participation in the project and direct the responsibility regarding other parts to the respective coauthor who wrote them 1. Situations in which the author of the manuscript did not participate in the project put at risk the image and credibility of the authors themselves.

The choice of the order of authors is very relevant after the manuscript is prepared. It seems prudent that the first (leading) author is the one with the greatest share in the entire work. From the second author, the order of participation tends to decrease. The senior advisor or researcher is usually the last author to be listed.

Petroianu 8 established a scoring table that facilitates the evaluation of the contribution of authors to the work. In an objective and measurable way, the author 8 proposes that each part of producing the work has a score that determines the order of the authors of the manuscript.

This type of quantitative method, explained prior to the start of the project, could avoid disputes regarding main authorship or the inclusion of coauthors who did not participate effectively in the work.

## PLAGIARISM AND SIMILARITY IN SCIENTIFIC WORK

Plagiarism is the action of presenting as one's own work artistic or scientific work that belongs to another. This practice is difficult to diagnose, considering that high textual similarity is usually the result of paraphrasing 9,10. Plagiarism is not only a faithful copy of a scientific paper but also the copying of a concept or idea included in a work without reference to the original.

These practices are very common problems found in the academic world, which has led several institutions to create guidelines to prevent these behaviors. The Scientific Electronic Library Online program (SciELO) 11 and the development agencies Foundation for Research Support of the State of São Paulo (FAPESP) 12, Coordination for the Improvement of Higher Education Personnel (CAPES) 13, and the National Council for Scientific and Technological Development (CNPq) 14, for example, have guidelines on plagiarism in academic work.

The CAPES 13 and SciELO 11 aim to use programs and applications to detect similarities in academic projects to avoid this behavior “that contaminates research, producing irreparable damage”.

Some sections such as the methods section are required to reproduce a work, and they may be present in other studies in a similar way, thus representing a similarity and not plagiarism. The CAPES 13 states that there should be a limit to these “recycled” parts of the work so that it may be accepted as an academic publication without harming the quality.

Self-plagiarism is another example of a complicated discussion that is also present in academia. Theoretically, self-plagiarism occurs when authors reuse their own material that is already published without reference to it 15. However, owning the intellectual property of the text published may render this question unfounded 16. In some situations, this feature is beneficial, as it brings to the public information that complements information described previously.

The Best Practices Guide for Strengthening Ethics in Scientific Publication from SciELO 11 and the CSE guidelines 10 instruct journal editors on how to proceed when misconduct is suspected.

## CONFLICT OF INTERESTS

The credibility of scientific information depends on the clarity and transparency with which conflicts of interest are presented 1,10,17. A conflict of interest is established when the professional principle, which focuses on the well-being and health of patients, and the commitment to the truth and integrity of research become dependent on an external variable, such as funding and personal issues 1.

According to the ICMJE 1, when authors submit articles, regardless of the type of article, they become responsible for declaring any financial or personal relationships that may be construed as bias in any way. In addition, this information should be explicit in the publication.

A great portion of journals provide specific terms for author's statements regarding conflicts of interest. Gollogly & Momen 18 argue that it is desirable for journals and their editors to act more proactively by standardizing the instructions regarding conflicts of interest for authors, so that journals participate in the process prior to submission, when the studies have already been completed.

## COPYRIGHTS, THEIR TRANSFER, AND THE OPEN ACCESS SOLUTION

Copyrights include the prerogative to present, disseminate, and distribute a particular material that is protected by authorship determinations 10,19. In most countries, there are laws defining how authorship and copyright issues are addressed. In the academic environment, many journals provide terms and documents proposing the transfer of the authorship of a scientific article to the publisher, which is then responsible for editing, hosting, or printing the article as well as providing a means to locate it, such as database indexing.

When published in peer-reviewed journals or as subscription access articles, the journal requires that the copyright for the article published be transferred to the publisher, with the exception of particular situations. The author retains the rights to the intellectual property developed, patents, and data collected, and only the edited file becomes the property of the journals 20.

In practice, the use of copyrighted articles is considered by some an obstacle to the advancement of science because it limits the possibility of reading them to those who have subscriptions or pay for articles on demand 16. In this sense, recent initiatives such as Sci-Hub and ResearchGate have acted forcefully for the free online availability of scientific papers from closed bibliographic databases through unilateral copyright infringement 21,22.

Thus, sharing scientific articles, which scientists have done for centuries, has become much easier, allowing studies to quickly cross oceans and continents, reaching destinations that no longer depend on publishers and indexers to be found.

Contrary to such copyright breach initiatives, a manifesto was drafted by a coalliance of scientific editors including the American Chemical Society, the American Medical Association, the American Physiological Society, Atlantis Press, BMJ, Brill, Elsevier, Future Science Group, IEEE, IWA Publishing, KeAi Publishing, Oxford University Press, Portland Press (wholly owned by the Biochemical Society), Wiley, Wolters Kluwer, and World Scientific Publishing. This manifesto was accompanied by active internet monitoring and removal requests for texts available on global computer networks 21.

In case authors wish to maintain their rights to the article in its final format with guaranteed free access to it, they may also choose a free access publication or open access 18. In this case, the advantages of free distribution and greater numbers of references to studies culminate in the payment of fees that correspond to the editorial process 18,20. The initiative of SciELO is among the main initiatives in this context 23.

While some journals work only with open or closed systems, several others allow authors to choose the type they wish to use 18,20. If authors choose open access, they assume the costs or remain in the closed system.

Thus, the evaluation of the selection criteria of authors should be well founded and performed so that everyone can list their participation in the text and respond consciously to all legal matters the work may entail.
